# Situs inversus with levocardia in a 15-year-old male adolescent: a case report

**DOI:** 10.1186/s13256-023-04254-9

**Published:** 2023-12-03

**Authors:** Telila Mesfin, Ibrahim Kedir, Teketel Tilahun, Kenbon Seyoum, Sisay Dadi, Neway Ejigu, Fikreab Desta, Girma Geta, Mesfin Tsegaye, Husien Salih

**Affiliations:** 1 School of Medicine, Goba General Hospital, Madda Walabu University, Goba, Oromia Ethiopia; 2 Department of Public Health, Menelik II Comprehensive Specialized Hospital, Finfinnee, Oromia Ethiopia; 3 Department of Midwifery, Goba General Hospital, Madda Walabu University, Goba, Oromia Ethiopia; 4 Department of Internal Medicine, Goba General Hospital, Madda Walabu University, Goba, Oromia Ethiopia; 5 Department of Public Health, Madda Walabu University, Goba General Hospital, Goba, Oromia Ethiopia; 6 Department of Nursing, Goba General Hospital, Madda Walabu University, Goba, Oromia Ethiopia

**Keywords:** Situs inversus, Levocardia, Situs inversus with levocardia, Adolescent

## Abstract

**Background:**

Situs inversus with levocardia is a rare anomaly in which the heart is present in the left chest but the abdominal viscera are transposed. It is caused by a single incomplete penetration of an autosomal recessive gene. It is unclear what exactly causes situs inversus with levocardia. Even if situs inversus can be identified following a comprehensive physical examination, it is now possible to validate the results and search for further information and pathologies since medical imaging is so widely accessible.

**Case:**

A 15-year-old Oromo male child from a remote area of Bale Zone presented to the Goba Referral Hospital’s medical emergency outpatient department complaining of periumbilical pain that had persisted for 4 months. He frequently came to our hospital and was admitted three times with the same problem. Objectively, there was tenderness over the left lower quadrant and periumbilical area. The sonographic evaluation discovered the transposition of the liver and spleen with cardiac apex on the left side. He received conservative treatment with ceftriaxone 1 g intravenous twice a day and metronidazole 500 mg intravenous for 5 days, and he went home improved.

**Conclusion:**

Isolated levocardia is a rare form of situs inversus in which the heart is in the traditional levo position while the abdominal organs are in the dextro position. What causes situs inversus with levocardia is unknown. Despite the fact that situs inversus can be diagnosed after a thorough physical examination, medical imaging has allowed us to confirm the findings as well as understand more about diseases. Due to the severity of an underlying heart defect, situs inversus with levocardia has a dismal prognosis.

## Introduction

Three categories—situs solitus (normal body asymmetry), heterotaxy, and situs inversus (mirror image orientation of abdominal and thoracic organs)—can be used to categorize the positions of the abdominal and thoracic organs [1]. Situs inversus totalis refers to the complete reversal of normal organ position, which includes both the thoracic and abdominal organs. Between the two extremes, situs solitus (normal) and situs inversus (reversed) totalis, is the spectrum of situs ambiguous (indeterminate), defined by Aylsworth as isomerism, heterotaxy, and numerous abnormalities in one or more thoracic or abdominal organs [2]. Situs inversus with levocardia is a rare anomaly brought on by a single imperfect penetration of an autosomal recessive gene in which the heart is present in the left chest but the abdominal viscera are transposed [3, 4]. According to Taussig’s book on congenital heart abnormalities, it appears infrequently [5]. It is estimated to affect 1% of all cases of congenital heart disease, with the majority of these cases being serious cardiac anomalies [6, 7]. It is estimated that a significant majority, reaching up to 95%, of instances involving isolated levocardia are accompanied by cardiac abnormalities, such as obstructions in the right ventricular outflow tract (RVOT), defects in the septum, the reversal of cardiac chambers, and the transposition of cardiac chambers [8]. Only 6% of patients with levocardia are still alive at the end of 5 years, and 75% die during the first year of life [9]. Diagnostic and treatment methods may be complicated by this condition. Practitioners of medicine, such as gastroenterologists, radiologists, and surgeons, typically have little experience with these patients due to their rarity [10]. Clinically, situs inversus is asymptomatic when it presents alone. However, when accompanied by other disorders, the inverted anatomical position of symptoms can make the diagnosis more difficult. The clinical manifestation of diffuse abdominal pain can be explained by the fact that the peripheral nervous system components are not transposed but the organs are [11].

## Case presentation

A 15-year-old Oromo male child who came from the rural area of Bale Zone and visited medical emergency outpatient department of Goba Referral Hospital presented with a complaint of periumbilical pain of 4 month duration. The pain worsened over the past 2 weeks. It was migratory and prominent on the left lower abdominal quadrant. Associated with this he had a history of loss of appetite and easy fatigability of the same duration. Additionally, he had a past record of experiencing a bout of watery diarrhea, occurring 3–4 times a day, persisting for a week. He was admitted to our hospital three times for the same complaints, despite visiting the hospital many times in between. The first visit was 3 years prior and he was diagnosed with appendicular mass and multiple regional and peritoneal lymphadenopathy. He was managed with ceftriaxone 1 g intravenous twice a day and metronidazole 500 mg intravenous three times a day for 5 days consecutively and discharged improved with cephalexin 500 mg orally twice a day for 5 days and metronidazole 500 mg orally three times a day for 5 days. A year prior, he presented with the same complaint and was managed the same way. Otherwise, he had no history of nausea or vomiting, no urinary complaint, no history of trauma to his abdomen, and no history, family or otherwise, of diabetes mellitus, hypertension, and cardiac illness.

**Fig. 1:**
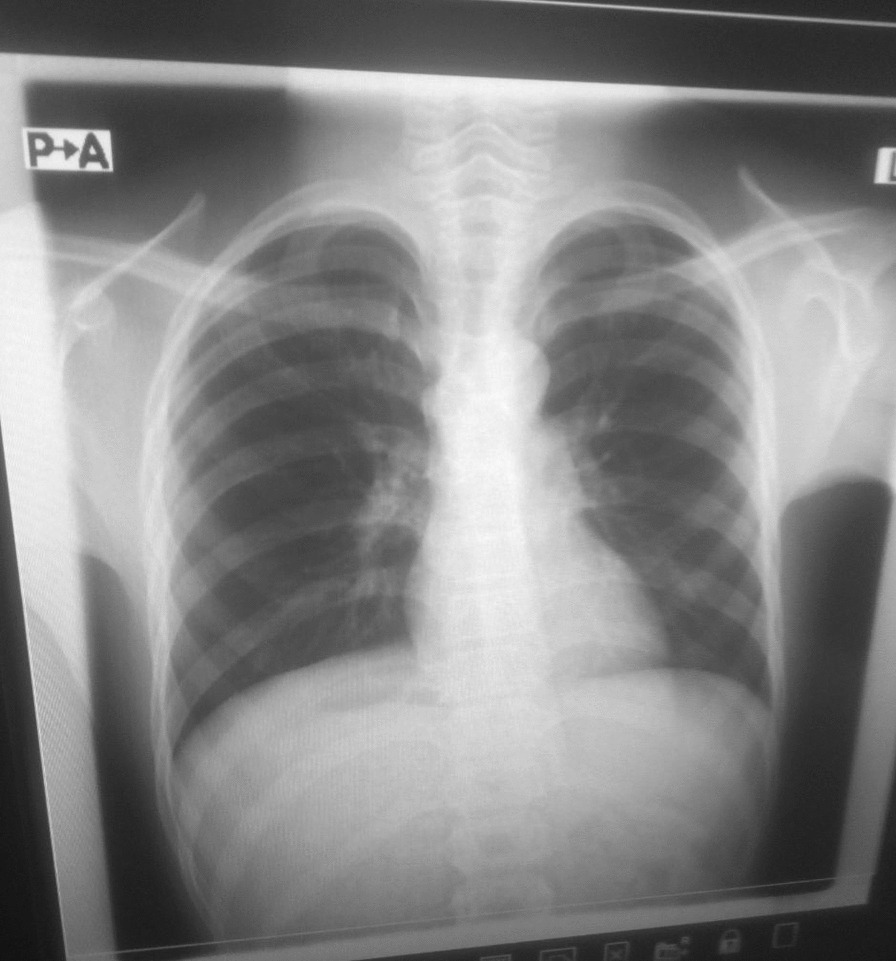
Posteroanterior chest x-ray showing cardiac apex positioned towards the left

Physical examination was unremarkable except for mild tenderness over left lower quadrant and periumbilical area. For this, he was investigated with hematological tests and imaging. Likewise, hemoglobin was 12.1 g/dl, hematocrit 37.5%, white blood cell 15,100/µl, neutrophil 45%, and lymphocyte 45%. Urinalysis and stool examination was non-revealing. Posteroanterior chest x-ray was normal (Fig. 1).

**Fig. 2:**
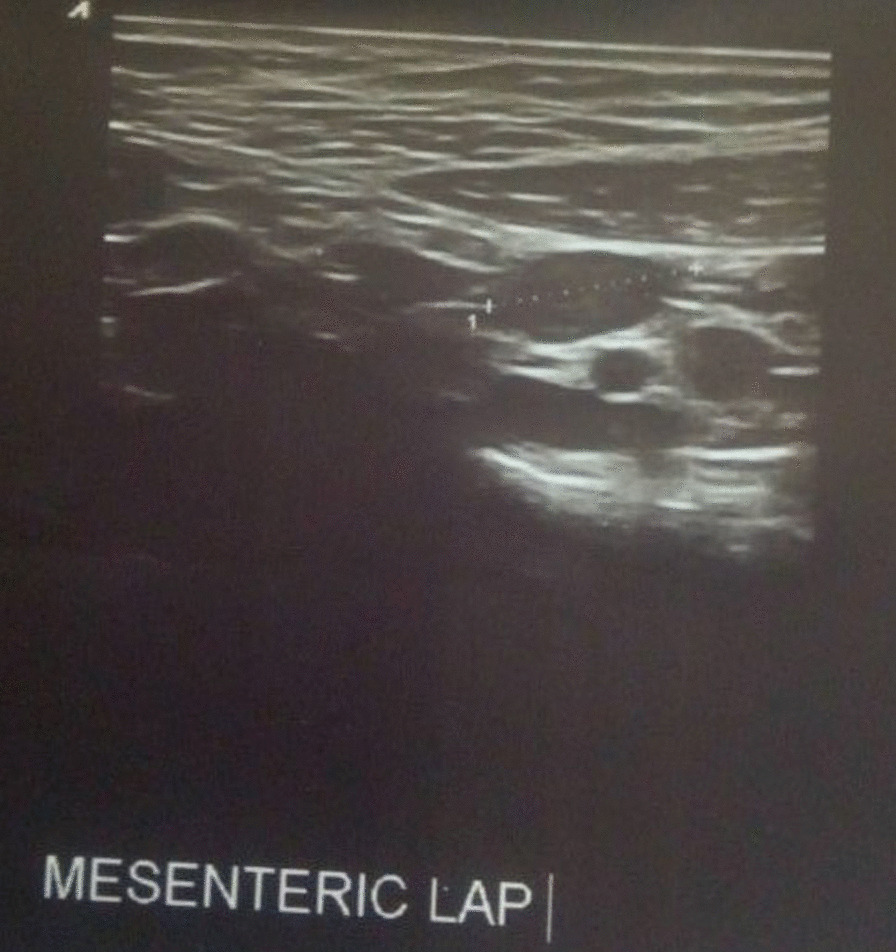
Abdominal ultrasound image showing multiple mesenteric lymphadenopathies

**Fig. 3:**
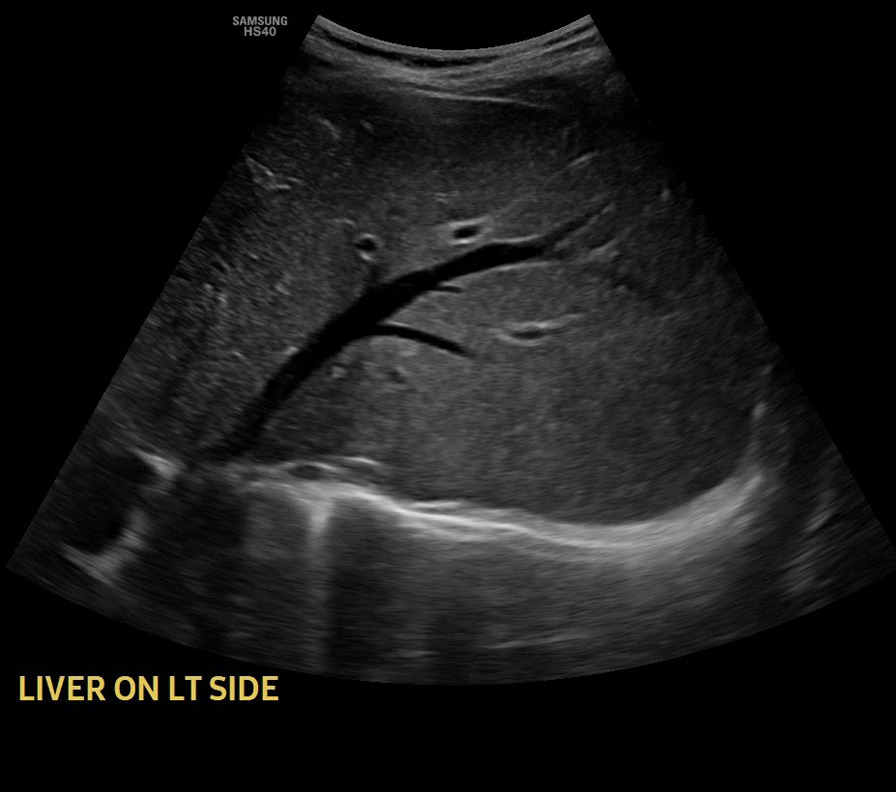
Sonographic picture of normal liver found on the left side

**Fig. 4:**
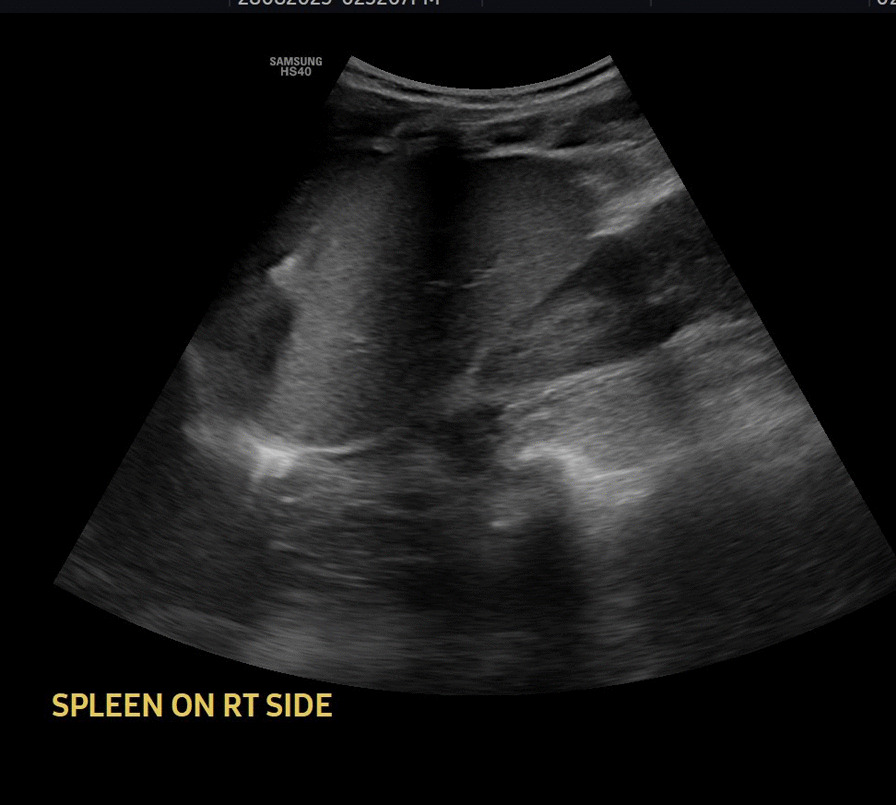
Sonographic picture of normal spleen situated on the right side

**Fig. 5:**
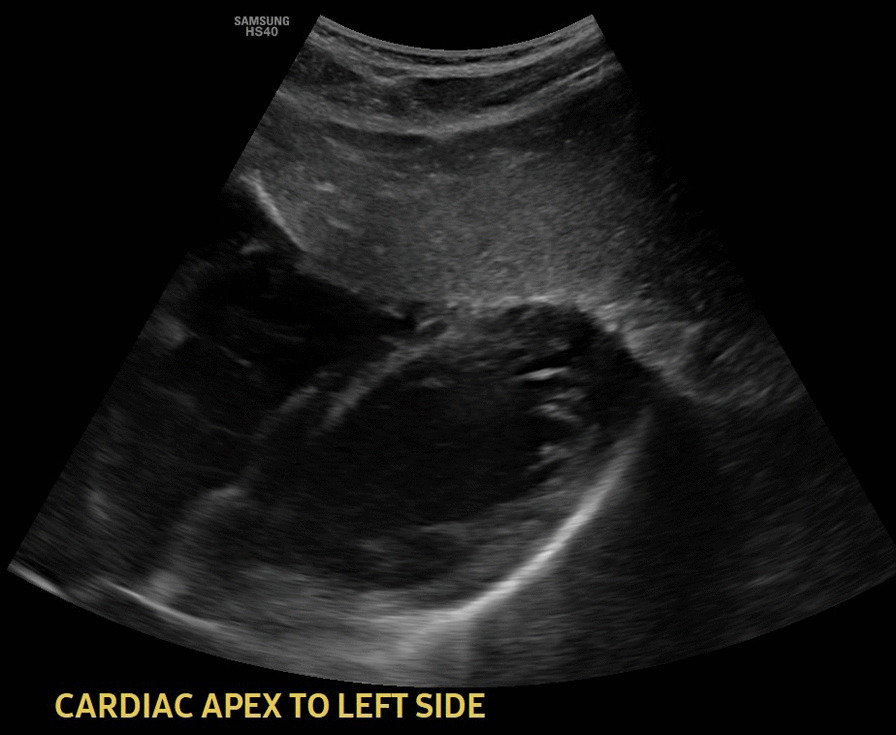
Ultrasound image of cardiac apex positioned toward the left

Abdominal ultrasound showed multiple regional and right peritoneal lymphadenopathies, the largest measuring 2.8 cm in size (Fig. 2). There was no loculated or free fluid collection in the peritoneal cavity. The liver was normal in size with homogeneous echo pattern and no focal lesion but found on the left side (Fig. 3). The spleen was normal in size and echo pattern but found on the right side (Fig. 4), and two-dimension echocardiography showed no structural or functional cardiac abnormalities. The heart apex was positioned toward the left side (Fig. 5).

He was eventually treated with ceftriaxone 1 g intravenous twice a day and metronidazole 500 mg intravenous three times a day for 5 days conservatively and discharged improved with cephalexin 500 mg orally twice a day and metronidazole 500 mg orally three times a day for 5 days. After 1 month of follow-up, we repeated sonographic evaluation. Mesenteric lymphadenopathies have shown significant remission.

## Discussion

The bodies of vertebrates have outward bilateral symmetry. As a result, it is remarkable that humans and all other vertebrates have internal asymmetry. Nearly all visceral organs are asymmetrical in structure and placement from left–right (L–R). Situs solitus refers to the proper placement of organs [12]. The final asymmetric organ configuration is influenced by two types of L–R asymmetry. Asymmetry of unpaired organs such as the heart, liver, spleen, stomach, small bowel, and colon is the first type. These organs’ embryonic primordia originate as midline structures and follow intricate patterns of movement, resulting in consistent location along the L–R axis. Some regions show a correlation between the development of asymmetry and the unilateral regression of initially bilaterally paired primordia, as shown during the development of the vascular system. The development of asymmetry in paired organs, notably the lungs, bronchi, and cardiac atrial appendages, is the second form of L-R asymmetry. These organs develop separate right and left forms, where right and left refer to morphology rather than location. No matter where they are along the L–R axis, a lung with three lobes is a right lung, whereas a lung with two lobes is a left lung. As a result, both paired and unpaired organs exhibit a consistent L–R asymmetry in the ultimate configuration of the organs. Furthermore, the asymmetry of paired and unpaired organs is directed or handed. This indicates that in all individuals of a species, the global asymmetric L–R organization is consistently orientated with respect to the anteroposterior and dorsoventral axes. Situs refers to the handed asymmetry, and situs solus refers to the natural organ placement [12, 13]. Malrotation, a congenital abnormality of the intestinal position, must always be distinguished from situs inversus. The foregut is the origin of the liver, bile ducts, gallbladder, esophagus, stomach, duodenum, and pancreas; these do not rotate during embryonic development. The midgut gives rise to the intestines, which rotate 270 degrees counterclockwise around the superior mesenteric artery to form the small intestine and colon up to the transverse colon. Malrotation happens when this method results in error. Malrotation is therefore unrelated to situs inversus [14, 15]. A rare form of situs inversus called isolated levocardia occurs when the heart is in the typical levo position, but the abdominal organs are in the dextro position. Van Praagh *et al*. have identified it as a unique entity that is not related to the situs ambiguous or heterotaxy (asplenia and polysplenia) syndromes [16]. In the general population, the estimated reported incidence is 1 per 22,000, while all patients with congenital cardiac disease have an incidence ranging from 0.4% to 1.2% [17, 18].

It is unclear what exactly causes situs inversus with levocardia [19]. One of the key developmental processes for asymmetric organs such as the heart, lungs, and digestive system is left–right (L–R) patterning. Delivery and interpretation of precise signals along the left–right axis are necessary for the proper development and positioning of internal organs. The basic heart tube eventually develops into either an l-loop or a d-loop, and the heart further differentiates by septation of the cardiac chambers and major blood vessels. An embryonic l-loop pivots into the right chest and should generate a mirror copy of the adult heart, but a d-loop is anticipated to lead to a typical situs solitus heart in the left chest. The L–R signaling system for the heart would ordinarily coordinate with a separate L–R signaling system for the orientation of the abdominal organs. It is unknown how the L–R signaling systems for the heart and the abdominal organs coordinate and exchange information. These aberrations may have been brought on by the two systems’ failure to coordinate normally through an unidentified process [19]. Though most of the patients with situs inversus usually have associated congenital heart diseases, our case has no such defect.

Despite the fact that situs inversus can be detected after a thorough physical examination, it is now possible to validate the findings and look for more information and pathologies thanks to the widespread availability of medical imaging. Diagnostic imaging methods that are more commonly used today include plain film x-ray and ultrasound (US). Dextrocardia, left-placed liver, and right-placed spleen are typical findings; polysplenia is a rare condition [20, 21]. To evaluate fine anatomical details and potential pathological findings, advanced imaging modalities such as computed tomography (CT) or magnetic resonance imaging (MRI) might be used. For establishing visceral organ position, heart apical position, intracardiac anatomy, and excellent vessel branching, CT offers good anatomic information. However, CT exposes patients to radiation, which is inappropriate for children. Cardiovascular magnetic resonance (CMR) is the best imaging technique for verifying the aforementioned information because it does not use ionizing radiation. The gold standard for measuring heart volumes and function is CMR [22–24]. We used posteroanterior (PA) chest x-ray and ultrasound imaging to diagnose the condition. It was an incidental finding while investigating the patient for abdominal complaints as is true for most of the reported cases so far.

Situs inversus with levocardia has a terrible prognosis; approximately 5–13% of patients live for more than 5 years, primarily because of the severity of a related cardiac defect. However, literature has reported prolonged survival of a patient with the condition as long as the age of 73 years [17]. This happens if there are no concomitant congenital heart diseases. The subsequent occurrences of bowel obstruction from midgut volvulus induced by concurrent intestinal malrotation may also be life-threatening [17, 18, 25]. Due to their complicated congenital heart abnormalities, the majority of patients with the heterotaxy syndromes die in childhood [26].

## Conclusion

Isolated levocardia is an uncommon variant of situs inversus in which the heart is in the conventional levo position while the abdominal organs are in the dextro position. It is unknown what causes situs inversus with levocardia. Despite the fact that situs inversus can be diagnosed after a comprehensive physical examination, medical imaging has made it possible to corroborate the findings and seek more information and diseases. Situs inversus with levocardia has a poor prognosis, owing to the severity of an associated heart abnormality.

## Data Availability

All data and materials for this case report are available from the corresponding author upon reasonable request.

## Abbreviations

BID: Two times per day
CMR: Cardiovascular magnetic resonance
CT: Computed tomography
IV: Intravenous
L–R: Left–right
MRI: Magnetic resonance imaging
PO: Per os
TID: Three times per day
US: Ultrasound

## References

[1] Lee SE (2006). Situs anomalies and gastrointestinal abnormalities. J Pediatr Surg.

[2] Aylsworth AS (2001). Clinical aspects of defects in the determination of laterality. Am J Med Genet.

[3] Young MD, Griswold HE (1951). Situs inversus of the abdominal viscera with levocardia: report of eight cases submitted to the Blalock-Taussig operation. Circulation.

[4] Zilberstein B (2000). The treatment of portal hypertension by videolaparoscopy in situs inversus totalis. Hepatogastroenterology.

[5] Taussig H (1947). Congenital malforiiations of the heart.

[6] Campbell M, Forgacs P (1953). Laevocardia with transposition of the abdominal viscera. Br Heart J.

[7] Doliopoulos T, Maillet J (1952). Left sidedness of the heart with inversion of the abdominal viscera; presentation of 5 cases. Cardiologia.

[8] Winer-Muram HT (1995). Adult presentation of heterotaxic syndromes and related complexes. J Thorac Imaging.

[9] Harris TR, Rainey RL (1965). Ideal isolated levocardia. Am Heart J.

[10] Blegen H (1949). Surgery in situs inversus. Ann Surg.

[11] Patel RB, Bhadreshwara K, Hukkeri S (2013). Laparoscopic appendicectomy in a patient with situs inversus totalis. Indian J Surg.

[12] Peeters H, Devriendt K (2006). Human laterality disorders. Eur J Med Genet.

[13] Ivemark BI (1955). Implications of agenesis of the spleen on the pathogenesis of conotruncus anomalies in childhood; an analysis of the heart malformations in the splenic agenesis syndrome, with fourteen new cases. Acta Paediatr.

[14] Sadler TW (2022). Langman’s medical embryology.

[15] Adams SD, Stanton MP (2014). Malrotation and intestinal atresias. Early Human Dev.

[16] VanPraagh S. Cardiac malpositions with special emphasis on visceral heterotaxy (asplenia and polysplenia syndrome). Nadas Pediatr Cardiol. 1992.

[17] Vijayakumar V, Brandt T (1991). Prolonged survival with isolated levocardia and situs inversus. Clevel Clin J Med.

[18] Budhiraja S (2000). Neonatal intestinal obstruction with isolated levocardia. J Pediatr Surg.

[19] Jo DS, Jung SS, Joo CU (2013). A case of unusual visceral heterotaxy syndrome with isolated levocardia. Korean Circul J.

[20] Haththotuwa H, Dubrey S (2013). A heart on the right can be more complex than it first appears. BMJ Case Rep.

[21] Suthar T (2012). Splenic infarct with polysplenia syndrome and situs inversus. Case Reports.

[22] Kashiwagi S (2013). Laparoscopic adrenalectomy in a patient with situs inversus. JSLS J Soc Laparoendosc Surg.

[23] Yoo S-J, Kim YM, Choe YH (1999). Magnetic resonance imaging of complex congenital heart disease. Int J Cardiac Imag.

[24] Bartram U, Fischer G, Kramer HH (2008). Congenitally interrupted inferior vena cava without other features of the heterotaxy syndrome: report of five cases and characterization of a rare entity. Pediatr Dev Pathol.

[25] Pickhardt PJ, Bhalla S (2002). Intestinal malrotation in adolescents and adults: spectrum of clinical and imaging features. Am J Roentgenol.

[26] Gayer G (1999). Polysplenia syndrome detected in adulthood: report of eight cases and review of the literature. Abdom Imaging.

